# The effect of interprofessional game-based learning on perceived cognitive load and self-efficacy in interprofessional communication and collaboration in patient safety incidents

**DOI:** 10.1371/journal.pone.0321346

**Published:** 2025-04-23

**Authors:** Azam Hosseinpour, Fatemeh Keshmiri

**Affiliations:** 1 Department of Operating Room, Faculty of Paramedical, Qom University of Medical Sciences, Qom, Iran; 2 Student Research Committee, Education Development Center, Shahid Sadoughi University of Medical Sciences, Yazd, Iran; 3 Medical Education Department, Education Development Center, Shahid Sadoughi University of Medical Sciences, Yazd, Iran; 4 The National Agency for Strategic Research in Medical Education, Tehran, Iran; Ataturk University, Faculty of Pharmacy, TÜRKIYE

## Abstract

**Background:**

This study aims to investigate the effect of the interprofessional game-based learning method on students’ perceived cognitive load and self-efficacy in interprofessional communication and collaboration during patient safety incidents, in comparison to traditional instructional methods.

**Methods:**

The quasi-experimental study was conducted in 2023–2024. The educational objective was to improve the interprofessional collaboration of the students in patient safety incidents. Students in operating room nursing and anesthesia nursing (n = 60) participated in this study. Interprofessional game-based learning and traditional methods were used in the intervention group and the control group, respectively. Participants completed two questionnaires about cognitive load and self-efficacy two months after intervention. The data were analyzed using descriptive tests (mean, standard deviation, and percentage) and analytical tests (including ANCOVA and Student T-test).

**Results:**

The IP-GBL intervention significantly improved students’ self-efficacy in the intervention group compared to the control group (F = 26.51, df = 1.57, p-value = 0.0001, Partial Eta Squared = 0.31). The IP-GBL method enhanced GCL and eliminated ECL and ICL compared to the traditional method. (p = 0.0001).

**Conclusion:**

The IP-GBL enhanced germane cognitive load and decreased the intrinsic and extrinsic cognitive load, which facilitated students’ learning. Patient safety training using interprofessional game-based learning has a favorable educational effect on students’ self-efficacy. The findings indicated that the students’ self-efficacy in interprofessional collaboration and communication regarding patient safety incidents in the surgical department significantly improved. Therefore, the interprofessional game-based learning method in formal and informal education of patient safety that requires collaboration between different professions is recommended.

## Introduction

The integration of game-based learning (GBL) into nursing education is an emerging pedagogical approach that is gaining increasing attention [1,2]. Game-based learning (GBL) is a pedagogical approach that utilizes game content and mechanics to achieve specific learning objectives, distinct from games designed primarily for entertainment. GBL and serious games are developed for educational purposes in contrast to gamification, which applies game elements in non-game contexts [3]. GBL by introducing elements of competition and challenge, provides opportunities for students to experience cooperative, engaging, and interactive learning [4,5]. The method assists students in retaining knowledge, developing reasoning and decision-making skills, and changing attitudes and behaviors through interactive and collaborative learning opportunities [6]. Integrating interprofessional strategy with GBL facilitates the development of crucial competencies such as teamwork, communication, challenge management, shared decision-making, and a deeper understanding of roles and responsibilities among team members in the game process [7]. IPE facilitates opportunities for students from diverse healthcare professions to learn with, from, and about each other, fostering effective collaboration within healthcare teams [7].

The design of the GBL method requires compliance with the principles of educational design and cognitive load management in the learning process [8–10]. Cognitive load, a key component in game-based learning design, refers to the burden placed on the cognitive system by a specific activity [11]. According to cognitive load theory, there are three types of cognitive load: Intrinsic Cognitive Load (ICL), Extraneous Cognitive Load (ECL), and Germane Cognitive Load (GCL) [11–13]. ICL arises from the complexity of educational materials and is influenced by factors such as individual skill level, the number of information elements, and the interaction between task elements. ECL is caused by the training format [14,15]. The theory of cognitive load highlights how inappropriate educational strategies can impose ECL, hindering learning. GCL refers to attempting to build and modify learning schemas and is influenced by factors such as motivation, effort, and inclusive metacognitive skills. [16]. Effective learning environment design aims to minimize Extrinsic Cognitive Load (ECL) and Intrinsic Cognitive Load (ICL) while promoting Germane Cognitive Load (GCL), thereby optimizing cognitive resources for meaningful learning and knowledge acquisition. Ineffective educational design in GBL can inadvertently increase cognitive load due to educational complexities, creating significant barriers to student learning. The cognitive load theory offers strategies to manage ECL and ICL and optimize GCL, for instance sorting the content from easy to difficult, providing worked examples, and using complete examples. These strategies help create a more conducive learning environment for students. After, the expansion of game-based learning in medical science education, studies recommended evaluating cognitive load to understand its impact on learner engagement and outcomes [17–19].

Operating room (OR) activities are characterized by complex, dynamic tasks marked by inherent variability and uncertainty, necessitating healthcare professionals to exhibit adaptability and precision in a high-pressure environment. A study by Henriques et al. (2016) identified several factors that compromise patient safety in the OR, including errors in surgical room setup, incomplete implementation of the nursing process, inadequate patient-provider communication regarding postoperative concerns, inadequate interprofessional collaboration, and non-adherence to the World Health Organization’s (WHO) recommended surgical safety checklist [20]. Despite the WHO’s recommendations and widespread institutionalization of the surgical safety protocol, adherence to this protocol often falls short of expected levels [20]. Human error and system failures are primary contributors to adverse events in the OR, highlighting the need for targeted education and training programs focused on patient safety. Furthermore, the rapidly evolving nature of surgical practices and technologies necessitates ongoing education and training for nurses to apply evidence-based practices [21,22].

Effective patient safety strategies, as identified by Amiri et al., include nursing education, effective communication, and collaborative relationships among healthcare professionals. Their findings emphasize the importance of promoting decision-making autonomy among nurses, managing conflicts, and implementing evidence-based standards of care. Additionally, the use of tools and interventions designed to reduce medical errors is crucial in enhancing patient safety. Moreover, critical topics such as teamwork, effective communication, surgical site verification, and medication safety are essential for error prevention and the promotion of a robust safety culture in the OR, and therefore, warrant emphasis in education and training programs. Notably, OR nurses and anesthesia nurses play a critical role in ensuring patient safety during surgical procedures, yet a significant gap exists in education and training for students in these professions regarding evidence-based practices that promote optimal patient safety in the OR [22].

The multifaceted nature of patient safety necessitates a comprehensive approach to education, one that enables students to develop capabilities, including identifying factors contributing to incidents, understanding the roles of different professions in managing patient safety risks, enhancing decision-making skills, and fostering interprofessional collaboration and communication. To address this complex issue, innovative educational approaches are required, incorporating simulation-based training, interprofessional collaboration, and just-in-time learning to equip OR and anesthesia nurses with the knowledge and skills necessary to provide safe and high-quality care to patients undergoing surgery [20,22]. Wang’s findings showed that different professions exhibit distinct patterns in identifying patient safety risks, highlighting the importance of interprofessional education (IPE) in patient safety training. IPE facilitates students from diverse professions to learn about risk patterns and improve their patient safety risk management capabilities [23].

The integration of Game-Based Learning (GBL) and interprofessional education strategies offers a unique opportunity to create training scenarios that involve students from various professions working together in interprofessional teams [5,6,24,25]. This interprofessional GBL (IP-GBL) approach provides a realistic and immersive environment for students to learn about patient safety incidents and risks, promoting teamwork and collaboration in managing complex patient safety situations [26–28]. In addition to teamwork and communication, self-efficacy is a crucial concept in preparing students for the workforce, particularly within healthcare systems that are inherently team-based and complex [29]. Self-efficacy reflects an individual’s belief in their ability to perform tasks competently, maintain confidence, and persevere in the face of challenges. Self-efficacy enhances teamwork, improves management of challenging situations, and boosts situational awareness among workers in complex healthcare environments [29]. Furthermore, self-efficacy has been identified as a critical predictor of future career success. It is widely recognized as a fundamental component of motivation, playing a significant role in fostering student engagement, especially in challenging situations. Investigating students’ self-efficacy in such contexts can provide valuable insights into their future behaviors [29–31]. Rusdi et al. demonstrated that nurses’ self-efficacy significantly influences nursing outcomes, highlighting its importance in professional performance. Furthermore, self-efficacy was found to act as a mediating variable in the relationship between communication skills and nursing performance, further underscoring its pivotal role in healthcare settings [31]. Further studies were recommended to evaluate the effect of the incorporation of IPE and GBL on learning outcomes [5,32].

This study aims to investigate the effect of the interprofessional game-based learning method on students’ perceived cognitive load and self-efficacy in interprofessional communication and collaboration during patient safety incidents, in comparison to traditional instructional methods.

## Materials and methods

### Study design and setting

**Fig 1:**
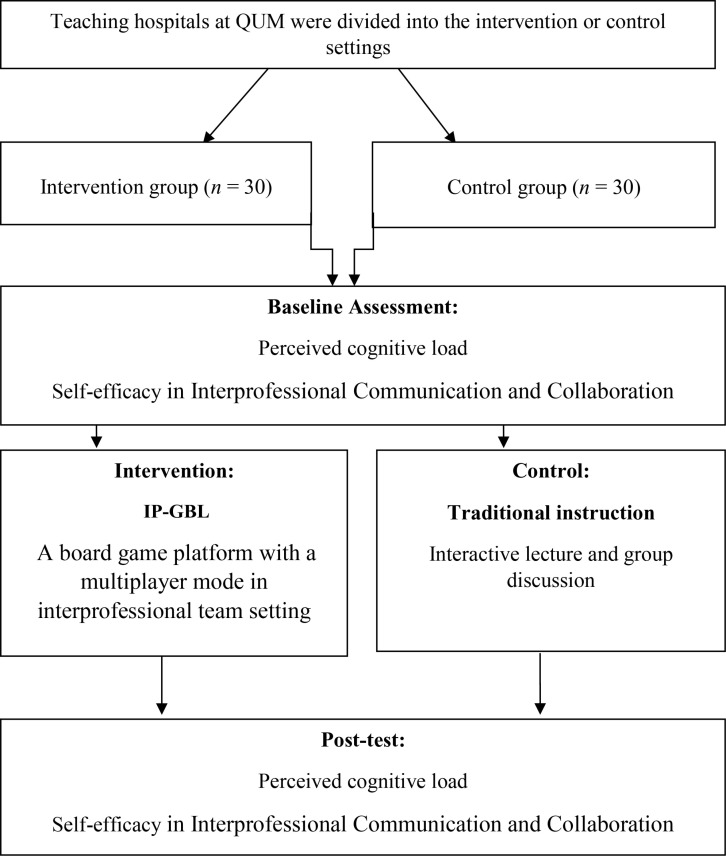
Flow Chart of the Study Steps.

The present study employed a quasi-experimental design and was conducted at Qom University of Medical Sciences in 2023–2024. (Fig 1).

#### Setting.

The current medical education curricula in the university are disciplinary-based. In addition, the dominant culture in the healthcare system of developing countries led to many barriers to the implementation of interprofessional educational programs [33,34]. Formal and informal curricula of different professions have overlooked the education of patient-centered care, team-based care, and patient safety.

In this context, professionals from various nursing specialties collaborate within surgical teams in the operating room. Their roles and responsibilities are outlined below, with a specific focus on the contributions of “anesthesia nursing” and “operating room nursing”.

Anesthesia nurses have undergone specialized education in anesthesia. They are authorized to independently induce, maintain, and conclude general anesthesia, as well as monitor patients receiving regional anesthesia in collaboration with an anesthesiologist. During anesthesia procedures, they are responsible for monitoring and assessing vital signs and parameters related to anesthesia depth, ventilation, circulation, temperature, and fluid balance. Moreover, they are expected to demonstrate nontechnical competencies, including teamwork, task management, situational awareness, and decision-making [35].

Roles of OR nursing: The three primary roles of Operating Room (OR) nursing are the scrub nurse, the circulating nurse, and the assisting nurse. Each role is integral to the surgical process, with responsibilities delineated across the preoperative, intraoperative, and postoperative periods. The scrub nurse, a sterile team member, prepares, passes, and manages surgical instruments, ensuring efficiency and safety during procedures. The circulating nurse, a non-sterile team member, coordinates OR activities, enforces protocols, reduces patient anxiety, and monitors for errors to maintain patient safety. This role also assists with patient positioning, performs equipment checks, and addresses sterility threats while supporting the surgical team. The assisting nurse provides instrumentation, handles specimens, documents medications, and communicates critical information during surgery. Postoperatively, the assisting nurse ensures instrument completeness, performs patient care tasks, and prepares the patient for transfer. Each role demands specialized skills, situational awareness, teamwork and strict adherence to protocols to ensure optimal surgical outcomes. These roles safeguard patient safety, enhance surgical efficiency, and maintain the integrity of the operative environment [36,37].

#### Participants.

Students in operating room nursing and anesthesia nursing who had worked in the surgical team in the surgical units and operating rooms were included in this study.

The inclusion criteria for the study required participants to have a minimum of 6 months of experience in operating room settings. Participants who declined to provide informed consent or had previously completed patient safety training courses were excluded from the study. Formal and informal training programs for internship courses in the relevant professions were coordinated through a curriculum, which was uniformly implemented across all teaching hospitals, thereby ensuring parity and consistency in the educational experience for students. A comparison of the patient safety protocols in the ORs of the two teaching hospitals revealed no significant differences in their implementation, suggesting that the protocols were comparable and consistent across the two settings.

The sample size was calculated as 60 students based on a Type I error of 0.05, a Type II error of 80%, and an effect size of 0.5, (30 students in each group). The students in two teaching hospitals affiliated with Qom University of Medical Sciences were assigned as the intervention and control groups. Participants were assigned to the intervention or control groups based on the teaching hospital where they studied their internship courses.

### Educational intervention

#### The educational objective of the interventions.

The educational program aims to enhance the preparedness of students to manage patient safety incidents in surgical units by recognizing roles and responsibilities within a surgical team and improving students’ self-efficacy in interprofessional collaboration and communication.

#### Educational content.

The educational content for both groups was developed based on patient-centered communication, interprofessional collaboration, patient safety, commonly encountered risks, and errors in operating rooms and surgical departments. This content was derived from a comprehensive review of the literature and WHO reports and guidelines [38–40]. The game scenario incorporated patient safety incidents with interaction and collaboration with peers, and patients, aligning with patient-centered principles [38–41]. Educational interventions were implemented using the game-based learning method in the intervention group and the traditional method for the control group. The course planning was undertaken by internship directors from the respective departments.

#### Duration of educational interventions.

The training interventions for both groups were conducted monthly over six months. (A total of six sessions). Each session lasted approximately 120–150 minutes.

### Educational intervention steps

#### Step 1: Design of game-based learning (GBL) intervention.

The game-based learning (GBL) intervention employed a puzzle-based format, utilizing a board game platform with a multiplayer mode, designed for an interprofessional team setting. The game’s design incorporated key elements, including motivational elements (solution and reward mechanisms, such as points and leaderboards), interactive elements (dilemmas and conflicts), and fun elements (story, challenge, and feedback). The IP-GBL platform was developed to incorporate problem-based scenarios, fostering a stimulating and interactive learning environment that promoted active engagement among students. Furthermore, the IP-GBL development integrated cognitive load strategies and the four key elements of game-based pedagogy [42–44].

*Theory-informed elements in IP-GBL design*: The development of the IP-GBL program was informed by the principles of Cognitive Load Theory, Interprofessional Education, and the four core elements of game-based pedagogy, which were integrated to create an innovative and effective learning experience.

Cognitive load theory played a pivotal role in informing the instructional design of the GBL intervention [42]. The game-based learning experience was designed using Cognitive Load Theory strategies, with a deliberate focus on optimizing learner cognitive load. To achieve this, a multifaceted approach was employed, incorporating the isolation of individual elements, progressive content sorting from simple to complex, multimodal learning, and the strategic integration of variability and visualization techniques.

Following the principles of Cognitive Load Theory, the game matrices were designed in two formats: a partially completed format and a blank format requiring students to fill in all cells. This design facilitated active engagement and critical thinking as students navigated the matrices, focusing on risk management steps. To ensure a smooth introduction to the game process, a completed game matrix was presented during an orientation session as a worked example, illustrating the tasks and objectives of the game. The game scenario was carefully crafted to incorporate contextual features and patient safety risks, exposing learners to a diverse range of realistic patient safety issues. The game’s content was tailored to reflect the main and common risks of patient safety in surgical department and operating room environments, ensuring the relevance and applicability of the game.

The game’s design allowed students to encounter risky situations and explore corresponding management strategies. To optimize the learning curve, the complexity of patient safety incidents was gradually increased across educational sessions. The game matrices progressed from simple to complex cases, with each matrix focusing on a primary incident within a multifaceted patient safety scenario. This design encouraged students to concentrate on specific tasks while understanding their broader implications. Multimodal learning was facilitated through the strategic integration of written tools, informative videos, and illustrative pictures, effectively depicting the consequences of patient safety risks and medical errors, such as retained foreign objects or electrocautery burns. This multimodal approach aimed to engage learners and enhance their understanding of the complex relationships between patient safety risks, medical errors, and risk management strategies.

Moreover, the IP-GBL incorporated the four essential elements of game-based pedagogy: cognitive engagement, behavioral engagement, social engagement, and affective engagement [43,44]. Cognitive engagement was designed to stimulate higher-order thinking, promote the application of knowledge, and foster adaptability in response to dynamic game scenarios. Behavioral engagement was facilitated through active participation and critical problem-solving, guiding students to apply their skills and knowledge to game challenges. Social engagement was cultivated through meaningful interactions with peers and interprofessional collaboration, balancing collaborative and competitive learning. The board game format enabled the exchange of ideas and perspectives among team members from diverse professions, enriching the learning experience. Affective engagement was leveraged to tap into the emotional and motivational aspects of learning, rendering the game enjoyable and intrinsically rewarding. Each game scenario was carefully crafted to evoke curiosity and excitement, while the introduction of a reward system served to enhance motivation and foster a sense of friendly competition.

The content of IP-GBL includes key patient safety risks often encountered in the surgical department and operating room settings. These risks included foreign objects retained post-surgery, wrong blood transfusions, technical errors failed intubations, pressure ulcers during surgery, medication errors, equipment malfunctions, patient falls, performing the wrong procedure, operating on the wrong patient, wrong-side surgery, cautery burns, failure to adhere to hygiene and disinfection protocols, blood reservation inaccuracies, nerve damage, hemorrhage, and excessive bleeding. The content validity of the game scenarios was evaluated and confirmed by an expert panel consisting of 12 professionals from diverse healthcare professions, including OR nursing, surgical specialties, and anesthesia nursing.

Each game included a realistic scenario, and the corresponding game matrix comprehensively addressed the roles of team members, the identification and mitigation of patient safety risks, and ways to prevent and manage them. The learners were asked to identify errors in patient safety, determine their underlying causes, delineate the roles of different healthcare professions, and establish effective prevention strategies and risk management approaches in the GBL process.

#### Step 2: Intervention Implementation .

The step was conducted in two stages.

*Preparation Stage*: Before the training program, students in the intervention group participated in an orientation session to familiarize them with the IP-GBL method. In alignment with cognitive load strategies, students were introduced to a worked example of the board game during this session and practiced a game matrix to enhance their understanding of the game mechanics.

*IP-GBL implementation Stage*: In the IP-GBL sessions, interprofessional groups were formed, comprising five students from diverse fields, including anesthesia and OR nursing. Each session involved participating in two game matrices, with students working collaboratively to complete each part within the allotted time frame. At the commencement of each session, participants established their tasks and assigned roles within the game matrix. They began by reading the scenario and illustrating the roles of various team members, including the surgeon, anesthesiologist, scrub OR nurse, circulator OR nurse, and anesthesia nurse, in the scenarios of patient safety incidents. Subsequently, they identified the causes of hazards and incidents, explored risk management strategies, and proposed preventive measures to mitigate patient safety risks within the matrix. The game matrix was collaboratively completed by the interprofessional team members through active participation, discussion, and consultation of educational resources. A facilitator guided the working group throughout the GBL process, ensuring interprofessional interactions and the participation of all team members in the collaborative learning experience.

In the second part of the process, completed matrices were evaluated by peer groups, who provided constructive feedback to facilitate refinement and improvement. This peer review process enabled students to receive feedback on their work, identify areas for improvement, and revise their matrices accordingly. Following the completion of the matrices, an experienced OR nurse, serving as the facilitator, provided additional feedback, allowing students to correct and enhance their work. A reward system was implemented to incentivize students, with points awarded for each section completed, including the identification of patient safety risks, determination of causal factors, understanding of team member roles, and development of prevention and management strategies. The facilitators in IP-GBL were two instructors in OR and anesthesia nursing with 10 years ± 2 working experience.

***Control Group***: The training for the control group was designed using a traditional method. The students in this group participated in interactive lectures and group discussions focused on patient safety incidents in the operating room. They explored guidelines for patient safety in surgical departments and discussed examples of patient safety risks. Students shared their opinions and insights on cases related to patient safety risks and their management.

***Evaluation***: The cognitive load associated with the educational interventions was evaluated from the perspective of the participants using a questionnaire. Moreover, self-efficacy in interprofessional communication and collaboration was assessed through self-assessment by the students. The evaluation was conducted two months after the educational program.

### Study measures

The questionnaire on “self-efficacy in collaboration and interprofessional communication” contains 33 items and was developed based on the framework of IPEC interprofessional collaboration capabilities by Hagemeie et al. in 2014 [45]. Responses were measured on a Likert scale ranging from “completely disagree” (1) to “completely agree” (5). In a previous study, the validity and reliability of this questionnaire were confirmed in the investigated context (Cronbach’s alpha =  0.95, ICC =  0.90). The questions were categorized into four domains: effective communication with the patient (11 items), patient engagement (8 items), interprofessional teamwork (10 items), and interprofessional interaction (4 items) [46].

The cognitive load questionnaire, developed by Krieglstein et al. [47], consisted of 15 items in three domains: ICL (5 items), ECL(5 items), and GCL (5 items). The validity and reliability of this questionnaire had been previously confirmed, with Cronbach’s alpha values of 0.75, 0.76, and 0.86 for ICL, ECL, and GCL, respectively. Items were scored on a scale from 1 to 9, with minimum and maximum scores of 5 and 45 for each domain.

### Data analysis

Data was summarized using descriptive tests (mean, standard deviation, and percentage). The analytical tests including the T-test and Analysis of Covariance (ANCOVA) were used to compare the scores in two groups. Moreover, The ANCOVA assessed the association between students’ scores, adjusting for gender, profession, and age. Data was analyzed using IBM SPSS Statistics 26.0. The level of significance was considered 0.05. Partial eta-squared (η2) was employed for effect size calculations. Effect sizes were interpreted based on Cohen’s guidelines, where values of approximately 0.01, 0.06, and 0.14 represent small, medium, and large effect sizes, respectively [48]. All effects are considered as significant at p < .05.

### Ethics approval and consent to participate

This study was approved by the Ethics Committee at the National Agency for Strategic Research in Medical Education, Tehran, Iran. (IR.NASRME.REC.1402.064). The written consent forms were obtained from all participants.

## Results

60 students participated in the study, with equal representation from OR nursing (n = 30, 50%) and anesthesia nursing (n = 30, 50%). The participants comprised 26 men (43.3%) and 34 women (56.7%), with a mean age of 21.70 years (SD =  1.01). All students were 4^th^ academic year and studied their internship course.

The cognitive load scores of the two learning methods are reported in Table 1. The results indicated that the GCL in the IP-GBL method was significantly higher than that of the traditional method (p = 0.0001). Additionally, the ECL and ICL in the IP-GBL method were significantly lower compared to the traditional method (p = 0.0001).

**Table 1:** Cognitive load scores in the IP-GBL and traditional methods in the intervention and control groups.

Cognitive load type	Educational method	Mean	Std. Deviation	Sig
ICL*	IP-GBL	12.10	2.95	0.0001
	Traditional method	16.80	4.03	
ECL**	IP-GBL	9.26	3.08	0.0001
	Traditional method	15.40	4.39	
GCL***	IP-GBL	35.76	4.71	0.0001
	Traditional method	24.06	4.11	

*Intrinsic Cognitive Load.

**Extraneous Cognitive Load.

***Germane Cognitive Load

The results revealed that the IP-GBL intervention significantly enhanced the self-efficacy of students in the intervention group compared to the control group, after adjusting the baseline scores, (F = 26.51, df = 1.57, p-value = 0.0001, Partial Eta Squared = 0.31). Table 1 shows the students’ self-efficacy scores in interprofessional collaboration and communication. No significant difference was found in the students’ self-efficacy scores based on gender (p-value =  0.2) or profession (p-value =  0.6) in the intervention and control groups. The results of the ANCOVA revealed even after adjusting for the age, gender, professions, and baseline score of the students as a covariance, the students’ self-efficacy scores in the IP-GBL in the intervention group were higher than the students’ scores in the control group (F (1.54) =  26.64, P =  0.0001, Partial Eta Squared =  0.33).

The results showed the educational effect of the educational intervention on the control group was reported at a large level in three domains of communication with the patient (F = 10.93, df = 1.57, p-value = 0.002, Partial Eta Squared = 0.16), the patient engagement (F = 20.15, df = 1.58, p-value = 0.001, Partial Eta Squared = 0.26) and the inter-professional interaction (F = 10.15, df = 1.57, p-value = 0.002, Partial Eta Squared = 0.15) by adjusting the baseline scores. The educational effect in the domain of interprofessional communication was at a medium level. (F = 4.14, df = 1.58, p-value = 0.04, Partial Eta Squared = 0.06). (Table 2)

**Table 2:** Students’ self-efficacy scores in interprofessional collaboration and communication in the intervention and control groups.

Domains	Groups	*Baseline score*			*Post-test score*		
		Mean	Std. Deviation	Sig	Mean	Std. Deviation	Sig
Communication with patient	Intervention	22.73	3.05	.376	35.70	3.71	.001
	Control	22.03	3.02		32.83	2.60	
Engagement with patient	Intervention	16.90	2.83	.881	26.53	2.50	.000
	Control	17.00	2.27		24.13	1.47	
Interprofessional teamwork	Intervention	21.60	3.66	.946	34.46	3.84	.002
	Control	21.53	3.97		31.56	3.10	
Interprofessional communication	Intervention	9.16	1.62	.657	13.70	2.58	.046
	Control	8.96	1.84		12.53	1.77	
Total	Intervention	70.40	6.32	.626	110.40	7.92	.000
	Control	69.53	7.35		101.06	5.80	

## Discussion

The results of the study demonstrated that the German cognitive load associated with the interprofessional game-based learning method, which facilitated effective learning, was significantly higher compared to the traditional method. In contrast, the extraneous and intrinsic cognitive loads associated with the game-based learning method were significantly lower than those associated with the traditional method. Notably, the findings indicated a large educational effect size of the IP-GBL intervention on students’ self-efficacy scores, suggesting that the IP-GBL intervention had a substantial and positive impact on students’ self-efficacy.

Game-based learning provides a valuable opportunity for students to engage in problem-solving through participatory and collaborative learning principles [49]. The primary objective of this study was to design an educational intervention that would enable students to engage with complex, real-world scenarios and develop decision-making skills in response to critical patient safety incidents within an interprofessional team context. To achieve this objective, a board game platform was developed, incorporating puzzle game elements that facilitated students’ comprehension of key concepts and error mitigation strategies through an interactive learning experience. The game’s design was intentionally structured to simulate the complexities of real-world patient safety challenges, requiring students to identify the root causes of errors, develop evidence-based solutions, and reflect on their learning through trial and error. Furthermore, the game’s multiplayer mode was designed to foster interprofessional collaboration and teamwork, enabling students to work together in teams to navigate the challenges and develop essential communication, problem-solving, and decision-making skills. The present results indicated that the IP-GBL method successfully enhanced GCL while eliminating ECL and ICL. Effective cognitive load management is essential for meaningful learning. The findings highlighted the successful management of cognitive loads in IP-GBL through interprofessional learning methods, team collaboration, and the progressive nature of challenges, from simple to complex. Hu et al. approved the impact of serious games on reducing cognitive loads, as it enables learners to better understand and organize their knowledge [50]. Consistent with the present study, Hu et al. found that computer games used in emergency training resulted in lower cognitive loads compared to traditional lectures. Further research is recommended to explore the impact of cognitive load on process and outcome factors associated with IP-GBL.

The results highlighted that creating an environment for interprofessional collaboration within game-based learning had a significant positive impact on students’ self-efficacy scores for interprofessional collaboration and communication. The study by Zou et al. found that game design that incorporated elements of collaboration and competition had a positive influence on student participation and learning outcomes and enhanced students’ self-efficacy [51]. Similarly, the present study incorporated collaboration through interprofessional teams and introduced competition through point systems within the game matrices. Consistent with Zou’s results, the current study also revealed an improvement in students’ self-efficacy following the game-based learning intervention. [51]. Topçu employed the Kahoot! Tool to design game-based learning sessions in the individual and team training sessions for nursing students. The game-based approach positively impacted motivation, self-efficacy, and group commitment toward achieving common goals. The game in group sessions encouraged peer learning and help-seeking behaviors, reinforcing collaboration and mutual support within the learning process. Moreover, collaborative learning in the GBL method reduced stress and worry about mistakes, fostering a deeper learning experience. The IP-GBL tasks stimulated the information-seeking and help-seeking behaviors of students, encouraging them to gather information from diverse disciplines and fostering group collaboration [52]. Moreover, the interprofessional group setting promoted effective teamwork and mutual support in completing the matrices. These situations positively influenced the improvement of students’ self-efficacy.

The results indicated that struggling with patient safety risks and explaining the outcomes of patient safety incidents enhanced students’ understanding of their role in interactions with patients, leading to improved self-efficacy in patient engagement and interaction. The educational effect of the IP-GBL method was particularly notable in the domains of ‘communication with patients’ and ‘engagement with patients’. The design of the diverse scenarios in the IP-GBL method intentionally exposed students to complex challenges, highlighting the importance of adhering to patient-centered principles in healthcare settings. Through a three-dimensional triangle framework, students were required to articulate the distinct roles and responsibilities of healthcare team members, including physicians, patients, and nursing teams. The tasks embedded within the patient safety challenges were designed to foster students’ understanding of a patient-centered approach, promoting students’ self-efficacy in interacting with and engaging patients in a meaningful way. Chang’s study, which utilized gamification and blended learning to teach patient safety, reported similar findings. Their quantitative results revealed higher scores in effectiveness and learning about patient safety in the gamification group. In addition, students acknowledged that learning through gamification improved their understanding of the values of collaboration and communication, leading to enhanced learning outcomes and motivation [53]. McCoy et al. further reinforced the effectiveness of digital scenario-based games as an innovative mechanism for teaching patient safety concepts and promoting interprofessional interactions among medical students. Their findings demonstrated that the patient safety interprofessional education module provided a valuable opportunity for students to learn safety concepts and practice interprofessional communication in small teams [54].

The findings revealed a favorable educational effect of IP-GBL in the domain of interprofessional collaboration for the intervention group compared to the control group. This domain encompassed components such as recognizing one’s role and the responsibilities of other team members, engaging in supportive communication, providing feedback, and exchanging knowledge and experiences. IP-GBL provided learners with a unique opportunity to understand the roles and capabilities of their peers within the context of simulated conditions. Participants in the IP-GBL encountered various challenges that required interprofessional collaboration, practiced task division, and experienced the benefits of interprofessional participation. These processes positively influenced the improvement of self-efficacy in interprofessional collaboration among students in the intervention group. Similar to the present study, Schmuck’s results revealed the positive impact of board game-based interprofessional education on the development of teamwork skills, self-awareness, and recognition of the responsibilities of other team members [55]. Likewise, Chang’s study, which utilized online game-based learning with question-and-answer strategies, reported students’ collaboration in the game process including observation/visualization, summarization, group discussion, and team effort to find answers had a positive impact on improving students’ self-efficacy [56].

The educational effect of interprofessional game-based learning on the scores of interprofessional communication domains was reported at a medium level. The findings indicate that the least change was observed in students’ self-efficacy scores in this domain. Interprofessional communication among surgical team members is affected by various factors such as cultural factors, organizational atmosphere, and individual factors that may influence the results. Furthermore, stereotypes, lack of preparation for interprofessional work, and lack of interprofessional training experience in the investigated context countries [33,34] may also affect the results. In line with our results, Stark et al. utilized a board game for interprofessional education. Their results showed that the board game did not affect students’ attitudes toward interprofessional education and interprofessional collaboration. These results were influenced by several factors, including a lack of familiarity with interprofessional education, students’ weak perception of interprofessional collaboration, and their lack of experience as team members during an epidemic [57]. Further qualitative studies suggest exploring the factors of interprofessional communication during patient safety incidents.

## Limitation

One of the limitations of this study was the small sample size and lack of randomization of participants, which may have limited the generalizability of the findings. Furthermore, the assessment of students’ self-efficacy relied on a self-reported tool, which was administered only before and after the intervention, without any long-term follow-up. This may have limited the ability to capture any sustained changes in self-efficacy over time. Furthermore, the results of this study may be context-specific, and their generalizability may be limited to similar educational settings with comparable interprofessional cultures and safety practices.

## Conclusions

Patient safety training utilizing interprofessional game-based learning has a favorable educational effect on students’ self-efficacy in interprofessional collaboration and communication. The educational intervention positively influenced students’ self-efficacy in domains of communication with patients, engagement with patients, and interprofessional collaboration in patient safety incidents. The educational effect on students’ scores in the domain of interprofessional communication was found to be at a medium level. Moreover, the IP-GBL enhanced germane cognitive load and decreased the intrinsic and extrinsic cognitive load, which facilitated students’ learning. Therefore, it is recommended that interprofessional game-based learning be incorporated into formal and informal education for patient safety issues that necessitate collaboration across different healthcare professions.
